# Clinical evaluation of oral fluid point-of-care testing for drugs of abuse compared to urinary point-of-care testing at a large-scale music festival

**DOI:** 10.1016/j.toxrep.2025.102192

**Published:** 2025-12-18

**Authors:** Frantzen MGM, Gresnigt FMJ, Litsenburg van RTH, Franssen EJF

**Affiliations:** a Researcher, Department of Hospital Pharmacy, OLVG, Amsterdam, the Netherlands; b Dutch Poisons Information Center, UMC, Utrecht 3508 GA, the Netherlands; c Event Medical Service, Loopkantstraat 2-E, Uden 5404 NB, the Netherlands; d Department of Clinical Pharmacy, OLVG Amsterdam, Oosterpark 9, Amsterdam 1091 AC, the Netherlands; e Emergency Department, OLVG Amsterdam, Oosterpark 9, Amsterdam 1091 AC, the Netherlands

**Keywords:** Point-of-care testing (POCT), Oral fluid drug test, Urine drug test, Drugs of abuse, Festival setting

## Abstract

**Background:**

Urinary point-of-care testing for recreational drugs is commonly used in clinical settings. An oral fluid-based point-of-care test is a less invasive alternative, but the reliability and clinical applicability in a real-life acute care setting is unclear.

**Aim:**

To assess the concordance of oral fluid point-of-care testing compared to urine point-of-care testing for recreational drugs in a prehospital clinical setting.

**Methods:**

This study was conducted during a large-scale dance music festival in October 2023. Urine and oral fluid samples were collected at the event medical station from volunteers with a suspected drug intoxication. Participants aged 18 years and older were included if both samples were provided and at least one substance tested positive. The percentage of positive oral fluid test results per recreational drug were compared with those of the urine point-of-care test.

**Results:**

A total of 78 patients were included. For most drug substances, positivity rates were similar between the two test types. Methamphetamine/3,4-methylenedioxymethamphetamine was the only substance that showed significantly more positive results in oral fluid compared to urine (p < 0.001). For all other substances, the differences between the two tests were small, with slightly higher positivity rates (on average 3.9 %) detected in oral fluid.

**Conclusion:**

Oral fluid point-of-care testing shows potential in specific scenarios but requires further validation. It is a less invasive alternative to urine point-of-care testing for recreational drugs in a clinical setting. Nevertheless, it is important to consider the differences in test characteristics, such as detection window. Further research is needed to evaluate the reliability in other populations and settings.

## Highlights

• Saliva drug testing was evaluated in an acute clinical setting during a festival
• 78 patients with suspected drug intoxication provided both urine and saliva samples
• Detection rates were similar for most substances in saliva and urine tests
• Methamphetamine/MDMA was more often detected in saliva than in urine
• Saliva testing is a promising and less invasive alternative to urine drug testing

## Graphical Abstract

**Figure:**
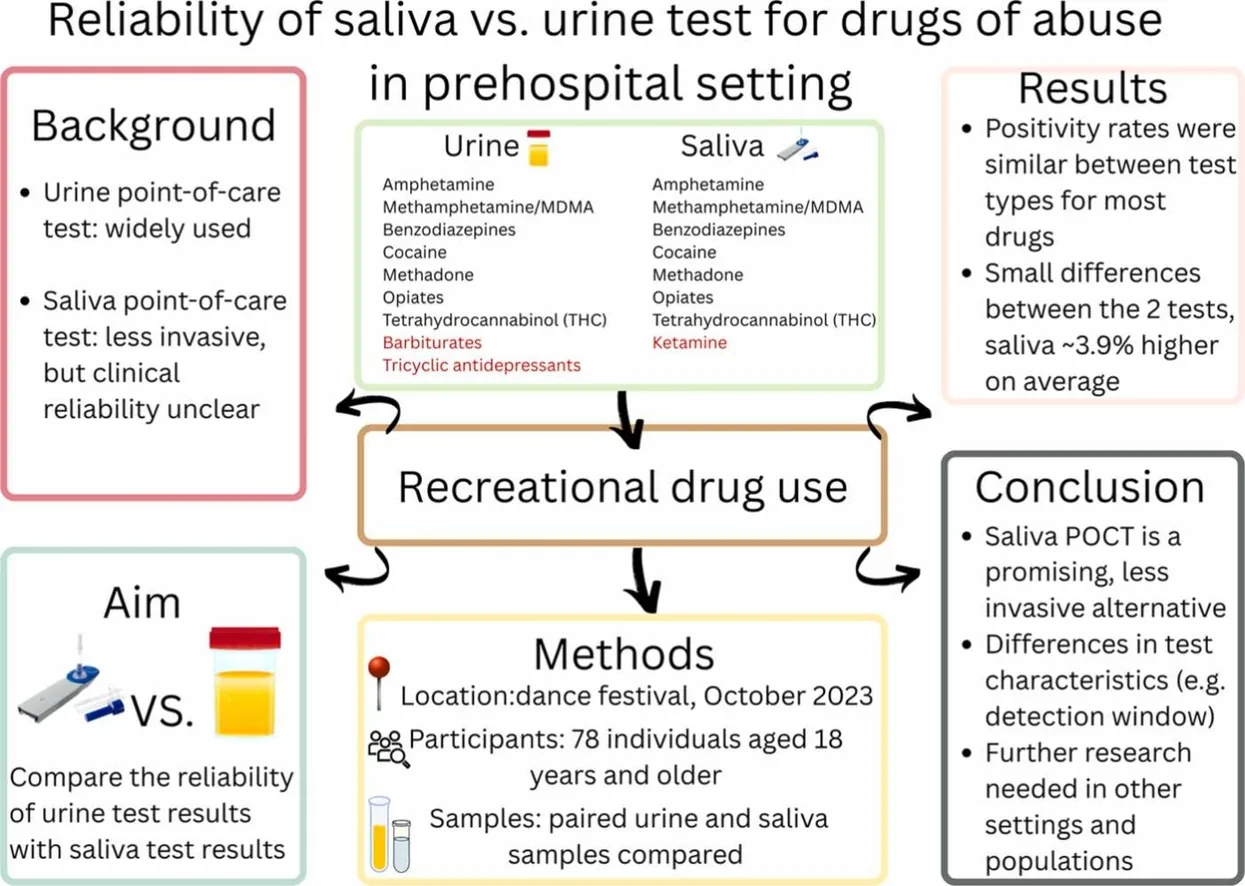

## 1 Introduction

In Europe, the prevalence of recreational drug use is high, with a notable increase in cocaine (+416 %), herbal cannabis (+260 %), methamphetamine (+135 %), and amphetamine (+42 %) between 2011 and 2021 [1], [2], [3]. The increase in recreational drug use has contributed to a rise in the number of patients with acute intoxications [1], [4], [5]. In an acute medical setting, it is important to know whether a patient’s clinical presentation can be explained by recreational drug use. If so, alternative treatment strategies are often required, and the use of certain medications must be avoided [5], [6], [7]. To ensure timely diagnosis, recreational drug point-of-care testing (POCT) in urine is commonly used [6], [8], [9]. The disadvantages of urine sample are the time-consuming nature of the collecting procedure, and the potential need for catheterization for individuals who are unable to urinate on their own. Recently, a POCT has become available to detect recreational drugs in oral fluid. Compared to urine, oral fluid contains more parent drug compounds and could provide more accurate information on recent recreational drug use [10]. Furthermore, oral fluid collection is non-invasive, faster, and has fewer biohazard risks compared to urine sampling.

No studies have addressed the potential of an oral fluid-based recreational drug test in a real-life acute clinical setting. Thus far the use of oral fluid testing has primarily focused on non-clinical settings, for example roadside screenings and workplace testing [11], [12]. These studies found that urine testing detected recreational drug use more frequently than oral fluid testing. In contrast, oral fluid testing is more indicative of an acute intoxication and therefore could be useful in certain scenarios, such as the detection of immediate intoxication. The employees stated that oral fluid testing was faster and more comfortable in general.

Therefore, this study aimed to assess the concordance of oral fluid point-of-care testing compared to urinary point-of-care testing for recreational drugs in a prehospital clinical setting.

## 2 Material and methods

### 2.1 Study design and setting

This prospective exploratory study was conducted at a first aid station of a dance festival organized during the Amsterdam Dance Event in October 2023, a multi-day electronic music festival in Amsterdam, the Netherlands. The study did not interfere with or influence the standard medical care.

Patients presenting at the first aid station with a suspected drug intoxication were eligible to participate in the study. Individuals were included if they were aged 18 years and older, provided both a urine and oral fluid sample, and had a positive result in at least one component of one of these tests. Patients were excluded if the oral fluid test was invalid.

### 2.2 Selection of participants

The study was conducted over two days, with attendance of approximately 15,000 visitors each day. On average, 60 individuals visited the first aid station per day, resulting in approximately 120 individuals visiting the aid station during the event. Of these visitors, 80 individuals were included in this study. A trained research team was present to carry out the study and actively approached visitors to participate. Individuals who presented at the first aid station without suspected drug use were nog tested and thus not eligible for inclusion in this study. The Medical Ethical Committee of OLVG declared that the study (W20.218) did not fall under the influence of the Medical Research Involving Human Subjects Act in the Netherland (Dutch WMO act). Written informed consent was undesirable to guarantee anonymity. After a brief explanation of the study, verbal consent was obtained. Each participant was assigned a unique code to ensure that the results of the urinary and oral fluid POCT were anonymized and could be matched. No patient data were obtained.

### 2.3 Procedure

Individuals entering the first aid station with a suspected drug intoxication, received the information letter and/or a brief explanation about the study. First, participants were asked to provide a urine sample in a small collection container. Subsequently, oral fluid was collected using the Drugtest 5000 STK oral fluid collector kits®. Participants performed the oral fluid test themselves, under supervision of trained research staff. The oral fluid test kit includes an indicator that turns blue once sufficient volume of oral fluid has been collected. After both samples were collected, participants were asked a few questions regarding their age, gender, substances used, and the reason for visiting the first aid station. A schematic overview of the study procedure is shown in Fig. 1.

**Fig. 1:**
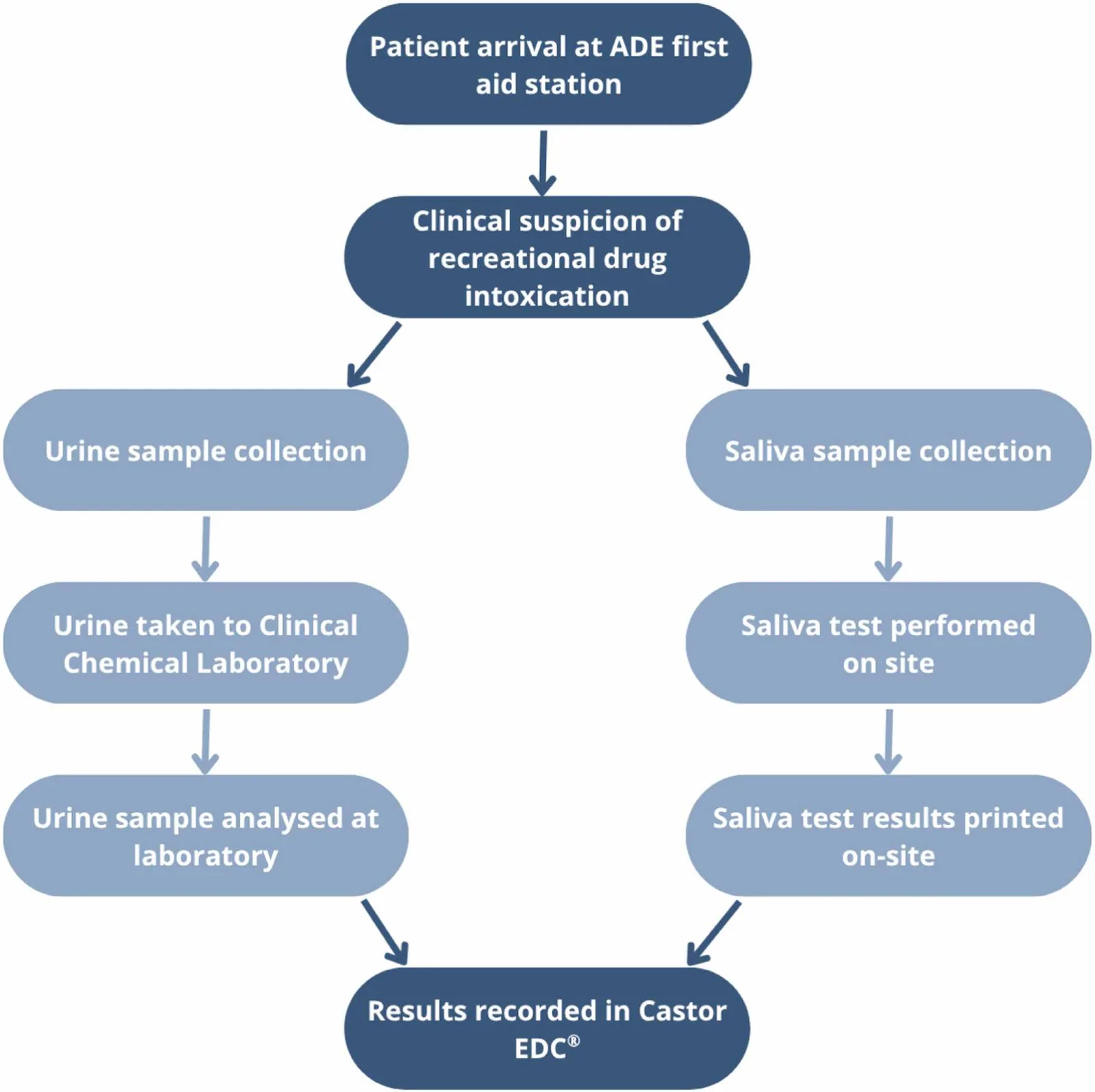
Schematic overview of the study procedure at the ADE first aid station, including sample collection and testing procedure.

### 2.4 Drug screening devices

Urine samples were analyzed using the Triage MeterPro® (Quidel, type QC, 999801, San Diego, CA, USA) device in combination with Triage TOX Drugs Screen® (TOX DS 94600) test strips. The Triage MeterPro® is a competitive immunofluorescence assay device for qualitative detection of parent drug compounds and metabolites [13], [14]. This urine POCT generates binary results (positive/negative) based on different predefined cut-off values specific for each substance.

Oral fluid samples were analysed using the Drugtest 5000 device® (Dräger, DT5000, Lubeck, Germany) and Drugtest 5000 STK oral fluid collector kits® [15]. The oral fluid collector kit contains a white indicator that turns blue when sufficient volume of oral fluid is collected. The Drugtest 5000 device® provides a qualitative result based on a three-point scale: positive, negative, and invalid. Prior to the start of the study, the device was validated in the pharmacy laboratory, according to current guidelines [16], [17], [18].

Both urinary and oral fluid POCT detect the native drug or metabolites of several recreational drugs, including amphetamine, methamphetamine, cocaine, benzodiazepines, opiates, methadone, and cannabis. The urinary POCT additionally detects barbiturates and tricyclic antidepressants, which are not included in the oral fluid POCT. Conversely, the oral fluid POCT detects the presence of ketamine, which is not included in the urinary POCT. The substance 3,4-methylenedioxymethamphetamine (MDMA) belongs to the group of methamphetamines. Furthermore, there are differences between the two tests in detection threshold and specificity (Appendix 1,2).

### 2.5 Data collection

Oral fluid samples were analyzed immediately on-site using two Drugtest 5000® devices. The results of the test were printed on-site, and participants were able to view their results. At the end of each study day, all urine samples were transported to the Clinical Chemical Laboratory of the OLVG hospital for analysis using the Triage MeterPro®. During the day, urine samples were stored at room temperature in well-sealed containers for a maximum of 8 h. After transport to the laboratory, samples were refrigerated and analyzed within 48 h. This storarage procedure is consistent with standard urine handling guidelines for POCT drug testing. Both urine and oral fluid samples were anonymized and linked using the same unique study code. The results of both tests were entered into Castor Electronic Data Capture (Castor EDC®).

### 2.6 Data analysis

All analyses were conducted using SPSS Statistics® version 29.0 – PS IMAGO PRO Data analysis. Descriptive statistics were reported using medians and interquartile ranges (IQR) for continuous data, and frequences and percentages for categorical variables. The oral fluid POCT results were compared with the urine POCT results, which served as the reference test. For each drug group, the percentage of positive tests in urine and oral fluid was compared using the McNemar test [19]. A p-value of P < 0.05 was considered statistically significant. In addition, the Cohen’s kappa values were calculated to assess the level of agreement between the urine and oral fluid POCT results for each drug group. A kappa value > 0.60 was interpreted using commonly applied thresholds: values < 0 indicate poor agreement, 0.00–0.20 slight agreement, 0.21–0.40 fair agreement, 0.41–0.60 moderate agreement, 0.61–0.80 substantial agreement, and > 0.8 almost perfect agreement.

The Medical Ethical Committee of the OLVG Hospital in Amsterdam approved the study, and the Medical Research Ethics Committee United (MEC-U) confirmed that this study was not subjected to the Dutch Medical Research Involving Human Subjects Act.

## 3 Results

### 3.1 Study characteristics

A total of 78 patients were included in the study. Of these patients, 38 (48.7 %) were male, and the median age was 24 years [range: 19–47 years old, IQR: 21–28; mean 25.2 years old, range 19–47]. Regarding the distribution of age, 44 (56.4 %) were aged younger than 24 years old. Two out of 80 oral fluid POCT were invalid (2.4 %), and these patients were excluded from the study. Of the urine samples, four urine POCT (4.9 %) showed no valid result during the first analysis; therefore, these urine samples were successfully re-analysed.

### 3.2 Main results

The number of positive urine and oral fluid POCT for each substance are shown in Table 1. The recreational drugs testing positive in urine and oral fluid POCT were methamphetamine/MDMA (75.6 % vs. 92.3 %), amphetamine (32.1 % vs. 35.9 %) and cocaine (11.5 %, 15.4 %), respectively. Barbiturates and tricyclic antidepressants could only be measured in urine; however, no positive test results were found. Ketamine could only be measured in oral fluid and was detected in 2.6 % (n = 2) of the oral fluid samples.

**Table 1:** Number of positive urine and oral fluid POCT results and comparison of detection rates per recreational drug type.

**Recreational drug type**	**Positive urine POCT** **n (%)**	**Positive oral fluid POCT** **n (%)**	**McNemar’s** **p-value**
Amphetamine	25 (32.1 %)	28 (35.9 %)	0.549
Methamphetamine/MDMA	59 (75.6 %)	72 (92.3 %)	**< 0.001**
Benzodiazepines	0 (0.0 %)	1 (1.3 %)	1.000
Cocaine	9 (11.5 %)	12 (15.4 %)	0.508
Methadone	0 (0.0 %)	0 (0.0 %)	-
Opiates	0 (0.0 %)	1 (1.3 %)	1.000
Tetrahydrocannabinol	5 (6.4 %)	5 (6.4 %)	1.000
Barbiturates	0 (0.0 %)	-	
Tricyclic antidepressants	0 (0.0 %)	-	
Ketamine	-	2 (2.6 %)	

Abbreviations: POCT: Point-of-Care Test; MDMA:3,4-methylenedioxymethamphetamine

A significant difference for methamphetamine/MDMA (p < 0.001) was found in favour of the oral fluid POCT. For all other recreational drugs, no significant differences were observed. Comparison of methadone was not possible, since no positive results were measured for urine or oral fluid. Overall, positivity rates were slightly higher in oral fluid testing compared to urine testing; amphetamine 3.8 %, methamphetamine/MDMA 16.7 %, benzodiazepines 1.3 %, cocaine 3.9 %, and opiates 1.3 %. On average, the difference between the urine and oral fluid POCT across all tested recreational drugs was 3.9 %, with the percentage of positive results consistently being higher in oral fluid.

Table 2 shows the level of agreement between urine and oral fluid POCT results per recreational drug, based on the Cohen’s kappa values. Substantial agreement was found for amphetamine (κ = 0.686) and THC (κ = 0.786). Cocaine (κ = 0.506) showed moderate agreement, while methamphetamine/MDMA (κ = 0.321) demonstrated fair agreement. Benzodiazepines (κ= −0.017) showed no meaningful agreement, and for methadone and opiates, kappa values could not be calculated due to the absence of positive test results.

**Table 2:** Cohen’s kappa and statistical agreement between urine and oral fluid POCT per recreational drug.

**Recreational drug type**	**Cohen's kappa (**κ)	**p-value**
Amphetamine	0.686	< 0.001
Methamphetamine/MDMA	0.321	< 0.001
Benzodiazepines	-0.017	0.870
Cocaine	0.506	< 0.001
Methadone	-	–
Opiates	-	–
THC	0.786	< 0.001

Abbreviations: MDMA:3,4-methylenedioxymethamphetamine

## 4 Discussion

This study evaluated the detection of recreational drugs comparing the oral fluid POCT results with urine POCT results, at the first aid station of a large-scale dance festival. Both test methods showed generally comparable positivity rates for the individual recreational drugs, however oral fluid POCT showed slightly higher positivity rates (on average 3.9 %) for several substances. A statistically significant difference was found for methamphetamine/MDMA, which also contained the largest positivity. For all other recreational drugs, no statistically significant differences were found between the two test methods. These results demonstrate that oral fluid POCT could be an alternative for urinary POCT in a real-life clinical setting.

Thus far, no research has been performed to evaluate the reliability and application of the oral fluid POCT in a clinical setting. A limited number of studies have been conducted in traffic or workplaces, which used different test devices [11], [12]. In one study, that assessed both urine and oral fluid POCT results, tetrahydrocannabinol was more frequently detected in urine samples than in oral fluid samples, which is not consistent with the results of this study, where tetrahydrocannabinol showed similar positivity rates in both tests [20]. However, that study used more complex analytical techniques, and sampling and testing was conducted in a workplace setting and not in a dance festival setting, where recent use is more likely. Besides this, the samples that were positive for tetrahydrocannabinol in the current study were small (n = 5). Since drug testing in a workplace or traffic setting differs from that in a clinical setting and the positivity rates of some of the drugs on the panel were quite small, additional research comparing urine and oral fluid POCT results is necessary to draw more definitive conclusions.

There were several differences between the urine POCT results and the oral fluid POCT results. This can be the consequence of 1) testing in different biological fluids (oral fluid versus urine) and 2) the actual compound that is picked up by the test (native drug and/or metabolites). Firstly, the fact that amphetamine, methamphetamine/MDMA, cocaine, and opiates were more frequently detected in oral fluid than in urine POCT, might be explained by differences in substance form detected and differences in sensitivity of both tests. While both oral fluid and urine POCTs are designed to detect relevant target compounds, differences in detection thresholds, sample matrix, and timing of sample collection may contribute to the observed variation in test results. For instance, in urine, THC can be detected up to four to six weeks after exposure, because the metabolite THC-COOH is detected by the urinary POCT. For cocaine, both the oral fluid and urine POCTs primarily target the metabolite benzoylecgonine, rather than the parent compound (see appendix 1). This also accounts for cocaine identification, where oral fluid POCT detects cocaine as native drug, and urinary POCT detects its inactive metabolite benzoylecgonine [21].

Secondly, oral fluid POCT testing is generally considered a better indicator of recent drug use than urine POCT testing, due to shorter detection windows and the presence of parent compounds in oral fluid [21]. Drug oral fluid concentrations correlate with blood concentrations [10], [22]. Urinary excretion of drug metabolites by the body needs time: i.e. formation of metabolites in the body followed by excretion into the urine. In the present study the samples were collected quite soon after recreational drug use. This can explain that methamphetamine/MDMA, amphetamine and cocaine were more frequently detected in oral fluid than in urine.

Further, the results of this study suggest that the reliability, the concordance and the applicability of the tests can depend on the type of recreational drug. Although the oral fluid POCT was more convenient to use in a clinical setting at a dance event, the results show that the reliability can vary depending on the type of recreational drug. This should be considered when using the oral fluid test as an alternative for the urinary test. Apart from methamphetamine/MDMA, the urine and oral fluid POCT showed similar results for the other substances. Although there were some small differences in the number of positive results, the two tests showed an overall agreement for the drug groups.

This study had some strengths and limitations. The strength is that in this study both urine and oral fluid samples of individual patients were simultaneously collected in a real-life acute clinical setting and by a well-trained team of care providers. Therefore it was possible to study the concordance of both tests in individual patients with a broad range of recreational drug intoxications. This study demonstrates that in these patients collecting oral fluid was less invasive, could be better controlled and was easier to perform as collecting urine. Nevertheless, 2.5 % of the oral fluid tests were invalid.

Conversely, there were several limitations that should be acknowledged. An important limitation is that both urine and oral fluid POCT are immunoassays, which are known to be susceptible to false positive and false negative test results [23]. Furthermore, the urine test was considered the reference test in this study. In an ideal situation, the test results should be compared with blood samples and tested with a Liquid Chromatography combined with mass spectrometry (LC-MS) technique. This method is more sensitive and specific than immunoassays. However, blood sampling in an acute setting at a dance event was not feasible due to ethical and logistical constraints.

In addition, the sample size in this study was relatively small for most recreational drugs, and the majority of positive test results were for methamphetamine/MDMA, which is a common recreational drug used at a dance event. This imbalance in the types of intoxications and the relatively small sample size may have limited the detection of differences for the other drugs groups. With a prevalence of more diverse intoxications and a larger sample size, different results might have been found, and more meaningful subanalyses for the less frequently used drugs would have been possible.

This skewed distribution is likely related to the type of event at which data were collected. Dance festivals, including the Amsterdam Dance Event (ADE), are known for high use of stimulant-type substances such as MDMA and amphetamine, while the use of depressants like benzodiazepines and opioids is much less common in these settings [24]. In addition, cannabinoids are used relatively infrequently at such events in the Netherlands. The observed pattern of drug positivity in our study likely reflects these usage trends [24].

Lastly, this study exclusively included participants who tested positive for at least one substance in either urine or oral fluid. This criterion was applied to ensure that the two tests could be compared. However, this limits the overall generalizability of the study results since these tests were only assessed in selected individuals who acknowledged recently used drugs. Individuals who denied recreational drug use were not tested, which limits the ability to evaluate the performance of the test in drug-negative individuals.

## 5 Conclusion

Oral fluid point-of-care testing for recreational drugs, compared to urine point-of-care testing, shows potential in specific scenarios but requires further validation. It is a less invasive alternative to urine point-of-care testing for recreational drugs in a clinical setting, and for amphetamine and methamphetamine/MDMA, the two tests produced roughly similar results. Nevertheless, it is important to consider the differences in test characteristics, such as detection windows. Further research is needed to evaluate the reliability in other populations and settings.

## Author contribution statement

MGM designed the study and wrote the manuscript. FMJ and EJF revised the manuscript.

## CRediT authorship contribution statement

**Frantzen MGM:** Writing – original draft. **Gresnigt FMJ:** Writing – review & editing. **Litsenburg van RTH:** Resources. **Franssen EJF:** Writing – review & editing.

## Ethics approval

The Medical Ethical Committee of the OLVG Hospital in Amsterdam approved the study, and the Medical Research Ethics Committee United (MEC-U) confirmed that this study was not subjected to the Dutch Medical Research Involving Human Subjects Act.

## Funding sources

This research did not receive any specific grant from funding agencies in the public, commercial, or not-for-profit sectors.

## Declaration of Competing Interest

The authors declare that they have no known competing financial interests or personal relationships that could have appeared to influence the work reported in this paper.

## Figures and Tables

**Table:** 

Dräger DrugTest 5000	Reference Sample contains [ng/mL]			
	< 14	14 – 20	20 – 26	> 26
positive	0	1	0	20
negative	98	4	0	2
Agreement: 97.5 %	GC-MS Cut-off: 14.0 ng/mL			

**Table:** 

Dräger DrugTest 5000	Reference Sample contains [ng/mL]			
	< 14	14 – 20	20 – 26	> 26
positive	3	4	0	27
negative	89	2	0	1
Agreement: 95.2 %	GC-MS Cut-off: 14.0 ng/mL			

**Table:** 

Dräger DrugTest 5000	Reference Sample contains [ng/mL]			
	< 24.5	24.5 – 35	35 – 45.5	> 45.5
positive	0	8	10	13
negative	90	2	0	0
Agreement: 98.4 %	GC-MS Cut-off: 24.5 ng/mL			

**Table:** 

Dräger DrugTest 5000	Reference Sample contains [ng/mL]			
	< 10.5	10.5 – 15	15 – 19.5	> 19.5
positive	2	1	1	16
negative	86	2	0	1
Agreement: 95.4 %	GC-MS Cut-off: 10.5 ng/mL			

**Table:** 

Dräger DrugTest 5000	Reference Sample contains [ng/mL]			
	< 3.5	3.5 – 5	5 – 6.5	> 6.5
positive	0	0	0	25
negative	63	1	0	6
Agreement: 92.6 %	GC-MS Cut-off: 3.5 ng/mL			

**Table:** 

Dräger DrugTest 5000	Reference Sample contains [ng/mL]			
	< 35	35 – 50	50 – 65	> 65
positive	1	1	10	9
negative	93	5	0	0
Agreement: 95.0 %	GC-MS Cut-off: 35.0 ng/mL			

**Table:** 

Dräger DrugTest 5000	Reference Sample contains [ng/mL]			
	< 14	14 – 20	20 – 26	> 26
positive	3	1	0	71
negative	60	0	0	1
Agreement: 97.1 %	GC-MS Cut-off: 14.0 ng/mL			

**Table:** 

Dräger DrugTest 5000	Reference Sample contains [ng/mL]			
	< 210	210 – 300	300 – 390	> 390
positive	2	9	4	22
negative	68	10	3	0
Agreement: 87.3 %	GC-MS Cut-off: 210.0 ng/mL			

**Table:** 

Drug name	Cut-off value (ng/mL)
Amphetamine	500
Methamphetamine/XTC	500
Barbiturates	200
Benzodiazepine	200
Cocaine	150
Methadone	100
Opiates	300
THC	50
Tricyclic antidepressant	1000

## Data Availability

Data will be made available on request.

## Appendix 1

Diagnostic specifics of saliva POCT by Drager 5000 DrugTest®: Detection limits and related tested compounds

### Cocaine

**Related compounds:** Benzoylecgonine (200 ng/mL), Cocaethylene (500 ng/mL), Cocaine (>20 ng/mL), Ecgoninemethylester (>10000 ng/mL), Norcocaine (400 ng/mL), Procaine (30000 ng/mL)

### Opiates

**Related compounds:** 6-Monoacetylmorphine (35 ng/mL), Codeine (25 ng/mL), Dihydrocodeine (20 ng/mL), Hydrocodone (20 ng/mL), Hydromorphone (10 ng/mL), Morphine (20 ng/mL), Morphine-3ß-D-glucuronide (10000 ng/mL), Nalorphine (35 ng/mL), Naloxone (1000 ng/mL), Norcodeine (8000 ng/mL), Normorphine (8000 ng/mL), Oxycodone (3000 ng/mL)

### Methamphetamine

**Related compounds:** Chloroquine sulphate (40000 ng/mL), Ephedrine (50000 ng/mL), Fenfluramine (300 ng/mL), MBDB (35 ng/mL), MDA (10000 ng/mL), MDEA (10000 ng/mL), MDMA (75 ng/mL), Procain (12 ng/mL), Propylhexedrine (100000 ng/mL), Pseudoephedrine (12 ng/mL), R-(+)-Methcatinone (4500 ng/mL), S-(-)-Methcatinone (7000 ng/mL), S(+)-Amphetamine (100000 ng/mL), S(+)-Methamphetamine (35 ng/mL)

### Benzodiazepine

**Related compounds:** 7-Aminoflunitrazepam (50 ng/mL), Alprazolam (10 ng/mL), Bromazepam (70 ng/mL), Chlordiazepoxide (3000 ng/mL), Clonazepam (15 ng/mL), Desalkylflurazepam (200 ng/mL), Diazepam (15 ng/mL), Flunitrazepam (20 ng/mL), Flurazepam (3500 ng/mL), Lorazepam (200 ng/mL), Midazolam (40 ng/mL), Nitrazepam (30 ng/mL), Nordiazepam (45 ng/mL), Oxazepam (40 ng/mL), Prazepam (9000 ng/mL), Temazepam (20 ng/mL), Triazolam (40 ng/mL)

### Tetrahydrocannabinol

**Related compounds:** Cannabidiol (90000 ng/mL), Cannabinol (350 ng/mL), delta8-THC (25 ng/mL), THC (delta9-THC) (5 ng/mL), THC-COOH (11-Nor-9-Carboxy-delta9-THC) (2 ng/mL), THC-OH (11-Hydroxy-delta9-THC) (10 ng/mL), CP 47,497 (>1000000 ng/mL), JWH-18 (>1000000 ng/mL)

### Amphetamine

**Related compounds:** Dopamine (40000 ng/mL), MBDB (>10000 ng/mL), MDA (100 ng/mL), MDEA (>10000 ng/mL), MDMA (100 ng/mL), Phentermine (5000 ng/mL), S(+)-Amphetamine (50 ng/mL), S(+)-Methamphetamine (>100000 ng/mL), Tyramine (5000 ng/mL)

### Methadone

**Related compounds:** Diphenhydramine (12000 ng/mL), EDDP (7000 ng/mL), LAAM (20 ng/mL), Methadone (1000 ng/mL), Pheniramide (1000 ng/mL)

### Ketamin

**Related compounds:** None

Reference: Dräger Safety AG & Co. KGaA. *Gebruiksaanwijzing Dräger DrugTest*® *5000 STK, 8-panel testkit*. Lübeck (DE): Dräger; 2016. Edition 03 – November 2016

## Appendix 2

Diagnostic specifics of urine POCT by Triage TOX Drug Screen®: detection limits

### Cut-off values

Reference: U.S. Food and Drug Administration. *510(k) substantial equivalence determination summary: Assay and instrument combination template* [Internet]. Silver Spring (MD): FDA; [cited 2025 Oct 22]. Available from: https://www.accessdata.fda.gov/cdrh_docs/reviews/k182719.pdf
